# Supplementary material for: Pyruvate Dehydrogenase Kinase 1 inhibition mediated oxidative phosphorylation enhancement in cartilage promotes osteoarthritis progression
Source: BMC Musculoskelet Disord. 2023 Jul 20;24:597. doi: 10.1186/s12891-023-06585-6 (PMC10357736; doi:10.1186/s12891-023-06585-6)
Supplement: Supplementary file 1 — Additional file 1. [file 12891_2023_6585_MOESM1_ESM.zip › MF.Gsea.1653627316242/GOMF_PRIMARY_ACTIVE_TRANSMEMBRANE_TRANSPORTER_ACTIVITY.html]

Details for gene set GOMF\_PRIMARY\_ACTIVE\_TRANSMEMBRANE\_TRANSPORTER\_ACTIVITY[GSEA]

|  || Dataset | 55457\_collapsed\_to\_symbols.cls.cls #OA\_versus\_normal.cls.cls #OA\_versus\_normal\_repos |
| Phenotype | cls.cls#OA\_versus\_normal\_repos |
| Upregulated in class | OA |
| GeneSet | GOMF\_PRIMARY\_ACTIVE\_TRANSMEMBRANE\_TRANSPORTER\_ACTIVITY |
| Enrichment Score (ES) | 0.41637674 |
| Normalized Enrichment Score (NES) | 2.0370784 |
| Nominal p-value | 0.0 |
| FDR q-value | 0.0062813694 |
| FWER p-Value | 0.062 |
Table: GSEA Results Summary

  

Fig 1: Enrichment plot: GOMF\_PRIMARY\_ACTIVE\_TRANSMEMBRANE\_TRANSPORTER\_ACTIVITY      
 Profile of the Running ES Score & Positions of GeneSet Members on the Rank Ordered List

  

| SYMBOL | TITLE | RANK IN GENE LIST | RANK METRIC SCORE | RUNNING ES | CORE ENRICHMENT || 1 | ATP6V1C1 | ATPase H+ transporting V1 subunit C1 [Source:HGNC Symbol;Acc:HGNC:856] | 17 | 1.125 | 0.0218 | Yes |
| 2 | UQCR10 | "ubiquinol-cytochrome c reductase, complex III subunit X [Source:HGNC Symbol;Acc:HGNC:30863]" | 53 | 0.987 | 0.0393 | Yes |
| 3 | NDUFA8 | NADH:ubiquinone oxidoreductase subunit A8 [Source:HGNC Symbol;Acc:HGNC:7692] | 67 | 0.961 | 0.0581 | Yes |
| 4 | ATP6V0D1 | ATPase H+ transporting V0 subunit d1 [Source:HGNC Symbol;Acc:HGNC:13724] | 135 | 0.872 | 0.0707 | Yes |
| 5 | NDUFS3 | NADH:ubiquinone oxidoreductase core subunit S3 [Source:HGNC Symbol;Acc:HGNC:7710] | 177 | 0.836 | 0.0847 | Yes |
| 6 | NDUFA1 | NADH:ubiquinone oxidoreductase subunit A1 [Source:HGNC Symbol;Acc:HGNC:7683] | 194 | 0.826 | 0.1004 | Yes |
| 7 | RALBP1 | ralA binding protein 1 [Source:HGNC Symbol;Acc:HGNC:9841] | 217 | 0.808 | 0.1153 | Yes |
| 8 | NDUFA13 | NADH:ubiquinone oxidoreductase subunit A13 [Source:HGNC Symbol;Acc:HGNC:17194] | 236 | 0.795 | 0.1302 | Yes |
| 9 | NDUFA10 | NADH:ubiquinone oxidoreductase subunit A10 [Source:HGNC Symbol;Acc:HGNC:7684] | 243 | 0.788 | 0.1460 | Yes |
| 10 | NDUFB6 | NADH:ubiquinone oxidoreductase subunit B6 [Source:HGNC Symbol;Acc:HGNC:7701] | 262 | 0.779 | 0.1605 | Yes |
| 11 | ATP6V1E1 | ATPase H+ transporting V1 subunit E1 [Source:HGNC Symbol;Acc:HGNC:857] | 287 | 0.758 | 0.1743 | Yes |
| 12 | UQCRC1 | ubiquinol-cytochrome c reductase core protein 1 [Source:HGNC Symbol;Acc:HGNC:12585] | 473 | 0.672 | 0.1736 | Yes |
| 13 | NDUFB3 | NADH:ubiquinone oxidoreductase subunit B3 [Source:HGNC Symbol;Acc:HGNC:7698] | 483 | 0.667 | 0.1866 | Yes |
| 14 | ATP6V1G1 | ATPase H+ transporting V1 subunit G1 [Source:HGNC Symbol;Acc:HGNC:864] | 515 | 0.655 | 0.1976 | Yes |
| 15 | NDUFS4 | NADH:ubiquinone oxidoreductase subunit S4 [Source:HGNC Symbol;Acc:HGNC:7711] | 531 | 0.647 | 0.2097 | Yes |
| 16 | ATP6V0E2 | ATPase H+ transporting V0 subunit e2 [Source:HGNC Symbol;Acc:HGNC:21723] | 532 | 0.647 | 0.2230 | Yes |
| 17 | NDUFA3 | NADH:ubiquinone oxidoreductase subunit A3 [Source:HGNC Symbol;Acc:HGNC:7686] | 550 | 0.641 | 0.2348 | Yes |
| 18 | COX5B | cytochrome c oxidase subunit 5B [Source:HGNC Symbol;Acc:HGNC:2269] | 585 | 0.628 | 0.2451 | Yes |
| 19 | NDUFA4 | NDUFA4 mitochondrial complex associated [Source:HGNC Symbol;Acc:HGNC:7687] | 624 | 0.613 | 0.2547 | Yes |
| 20 | COX6A1 | cytochrome c oxidase subunit 6A1 [Source:HGNC Symbol;Acc:HGNC:2277] | 653 | 0.605 | 0.2650 | Yes |
| 21 | ABCC6 | ATP binding cassette subfamily C member 6 [Source:HGNC Symbol;Acc:HGNC:57] | 729 | 0.587 | 0.2712 | Yes |
| 22 | ATP6V1D | ATPase H+ transporting V1 subunit D [Source:HGNC Symbol;Acc:HGNC:13527] | 750 | 0.581 | 0.2815 | Yes |
| 23 | NDUFA2 | NADH:ubiquinone oxidoreductase subunit A2 [Source:HGNC Symbol;Acc:HGNC:7685] | 878 | 0.549 | 0.2829 | Yes |
| 24 | NDUFB5 | NADH:ubiquinone oxidoreductase subunit B5 [Source:HGNC Symbol;Acc:HGNC:7700] | 902 | 0.542 | 0.2922 | Yes |
| 25 | NDUFC2 | NADH:ubiquinone oxidoreductase subunit C2 [Source:HGNC Symbol;Acc:HGNC:7706] | 929 | 0.532 | 0.3011 | Yes |
| 26 | NDUFA9 | NADH:ubiquinone oxidoreductase subunit A9 [Source:HGNC Symbol;Acc:HGNC:7693] | 943 | 0.529 | 0.3110 | Yes |
| 27 | NDUFB4 | NADH:ubiquinone oxidoreductase subunit B4 [Source:HGNC Symbol;Acc:HGNC:7699] | 968 | 0.525 | 0.3199 | Yes |
| 28 | NDUFB7 | NADH:ubiquinone oxidoreductase subunit B7 [Source:HGNC Symbol;Acc:HGNC:7702] | 1033 | 0.510 | 0.3254 | Yes |
| 29 | NDUFB1 | NADH:ubiquinone oxidoreductase subunit B1 [Source:HGNC Symbol;Acc:HGNC:7695] | 1055 | 0.506 | 0.3341 | Yes |
| 30 | NDUFS7 | NADH:ubiquinone oxidoreductase core subunit S7 [Source:HGNC Symbol;Acc:HGNC:7714] | 1071 | 0.501 | 0.3432 | Yes |
| 31 | NDUFC1 | NADH:ubiquinone oxidoreductase subunit C1 [Source:HGNC Symbol;Acc:HGNC:7705] | 1091 | 0.499 | 0.3520 | Yes |
| 32 | NDUFA7 | NADH:ubiquinone oxidoreductase subunit A7 [Source:HGNC Symbol;Acc:HGNC:7691] | 1097 | 0.496 | 0.3618 | Yes |
| 33 | COX8A | cytochrome c oxidase subunit 8A [Source:HGNC Symbol;Acc:HGNC:2294] | 1105 | 0.494 | 0.3714 | Yes |
| 34 | ATP1B1 | ATPase Na+/K+ transporting subunit beta 1 [Source:HGNC Symbol;Acc:HGNC:804] | 1177 | 0.481 | 0.3757 | Yes |
| 35 | ATP6V0E1 | ATPase H+ transporting V0 subunit e1 [Source:HGNC Symbol;Acc:HGNC:863] | 1207 | 0.474 | 0.3832 | Yes |
| 36 | NDUFS8 | NADH:ubiquinone oxidoreductase core subunit S8 [Source:HGNC Symbol;Acc:HGNC:7715] | 1208 | 0.474 | 0.3930 | Yes |
| 37 | NDUFS1 | NADH:ubiquinone oxidoreductase core subunit S1 [Source:HGNC Symbol;Acc:HGNC:7707] | 1229 | 0.471 | 0.4011 | Yes |
| 38 | ATP6V1H | ATPase H+ transporting V1 subunit H [Source:HGNC Symbol;Acc:HGNC:18303] | 1304 | 0.457 | 0.4047 | Yes |
| 39 | NDUFS6 | NADH:ubiquinone oxidoreductase subunit S6 [Source:HGNC Symbol;Acc:HGNC:7713] | 1417 | 0.441 | 0.4050 | Yes |
| 40 | NDUFV2 | NADH:ubiquinone oxidoreductase core subunit V2 [Source:HGNC Symbol;Acc:HGNC:7717] | 1642 | 0.407 | 0.3958 | Yes |
| 41 | NDUFV1 | NADH:ubiquinone oxidoreductase core subunit V1 [Source:HGNC Symbol;Acc:HGNC:7716] | 1663 | 0.404 | 0.4025 | Yes |
| 42 | ABCG1 | ATP binding cassette subfamily G member 1 [Source:HGNC Symbol;Acc:HGNC:73] | 1711 | 0.396 | 0.4070 | Yes |
| 43 | SURF1 | SURF1 cytochrome c oxidase assembly factor [Source:HGNC Symbol;Acc:HGNC:11474] | 1829 | 0.381 | 0.4056 | Yes |
| 44 | CYB561D2 | cytochrome b561 family member D2 [Source:HGNC Symbol;Acc:HGNC:30253] | 2010 | 0.360 | 0.3989 | Yes |
| 45 | COX7A1 | cytochrome c oxidase subunit 7A1 [Source:HGNC Symbol;Acc:HGNC:2287] | 2045 | 0.356 | 0.4036 | Yes |
| 46 | COX4I1 | cytochrome c oxidase subunit 4I1 [Source:HGNC Symbol;Acc:HGNC:2265] | 2167 | 0.342 | 0.4011 | Yes |
| 47 | NDUFS5 | NADH:ubiquinone oxidoreductase subunit S5 [Source:HGNC Symbol;Acc:HGNC:7712] | 2267 | 0.330 | 0.4002 | Yes |
| 48 | ATP12A | ATPase H+/K+ transporting non-gastric alpha2 subunit [Source:HGNC Symbol;Acc:HGNC:13816] | 2330 | 0.323 | 0.4019 | Yes |
| 49 | NDUFB2 | NADH:ubiquinone oxidoreductase subunit B2 [Source:HGNC Symbol;Acc:HGNC:7697] | 2460 | 0.307 | 0.3981 | Yes |
| 50 | COX7A2L | cytochrome c oxidase subunit 7A2 like [Source:HGNC Symbol;Acc:HGNC:2289] | 2517 | 0.302 | 0.3999 | Yes |
| 51 | NDUFB8 | NADH:ubiquinone oxidoreductase subunit B8 [Source:HGNC Symbol;Acc:HGNC:7703] | 2623 | 0.288 | 0.3976 | Yes |
| 52 | ABCB6 | ATP binding cassette subfamily B member 6 (Langereis blood group) [Source:HGNC Symbol;Acc:HGNC:47] | 2717 | 0.278 | 0.3961 | Yes |
| 53 | ATP2A1 | ATPase sarcoplasmic/endoplasmic reticulum Ca2+ transporting 1 [Source:HGNC Symbol;Acc:HGNC:811] | 2781 | 0.272 | 0.3967 | Yes |
| 54 | ATP6V0C | ATPase H+ transporting V0 subunit c [Source:HGNC Symbol;Acc:HGNC:855] | 2790 | 0.271 | 0.4016 | Yes |
| 55 | ABCC8 | ATP binding cassette subfamily C member 8 [Source:HGNC Symbol;Acc:HGNC:59] | 2844 | 0.266 | 0.4030 | Yes |
| 56 | COX6B1 | cytochrome c oxidase subunit 6B1 [Source:HGNC Symbol;Acc:HGNC:2280] | 2915 | 0.259 | 0.4028 | Yes |
| 57 | ABCG5 | ATP binding cassette subfamily G member 5 [Source:HGNC Symbol;Acc:HGNC:13886] | 2942 | 0.256 | 0.4060 | Yes |
| 58 | ATP2B2 | ATPase plasma membrane Ca2+ transporting 2 [Source:HGNC Symbol;Acc:HGNC:815] | 3023 | 0.247 | 0.4048 | Yes |
| 59 | ATP5F1B | ATP synthase F1 subunit beta [Source:HGNC Symbol;Acc:HGNC:830] | 3024 | 0.247 | 0.4099 | Yes |
| 60 | ABCD2 | ATP binding cassette subfamily D member 2 [Source:HGNC Symbol;Acc:HGNC:66] | 3031 | 0.246 | 0.4145 | Yes |
| 61 | NDUFS2 | NADH:ubiquinone oxidoreductase core subunit S2 [Source:HGNC Symbol;Acc:HGNC:7708] | 3072 | 0.243 | 0.4164 | Yes |
| 62 | ATP4B | ATPase H+/K+ transporting subunit beta [Source:HGNC Symbol;Acc:HGNC:820] | 3160 | 0.234 | 0.4144 | No |
| 63 | SLC4A1 | solute carrier family 4 member 1 (Diego blood group) [Source:HGNC Symbol;Acc:HGNC:11027] | 3233 | 0.227 | 0.4134 | No |
| 64 | ATP2B3 | ATPase plasma membrane Ca2+ transporting 3 [Source:HGNC Symbol;Acc:HGNC:816] | 3263 | 0.225 | 0.4157 | No |
| 65 | ABCC4 | ATP binding cassette subfamily C member 4 [Source:HGNC Symbol;Acc:HGNC:55] | 3344 | 0.217 | 0.4139 | No |
| 66 | COX7B | cytochrome c oxidase subunit 7B [Source:HGNC Symbol;Acc:HGNC:2291] | 3452 | 0.207 | 0.4098 | No |
| 67 | ABCA3 | ATP binding cassette subfamily A member 3 [Source:HGNC Symbol;Acc:HGNC:33] | 3549 | 0.198 | 0.4063 | No |
| 68 | ATP6V1G2 | ATPase H+ transporting V1 subunit G2 [Source:HGNC Symbol;Acc:HGNC:862] | 3709 | 0.184 | 0.3977 | No |
| 69 | COX15 | cytochrome c oxidase assembly homolog COX15 [Source:HGNC Symbol;Acc:HGNC:2263] | 3727 | 0.183 | 0.4001 | No |
| 70 | UQCRH | ubiquinol-cytochrome c reductase hinge protein [Source:HGNC Symbol;Acc:HGNC:12590] | 3827 | 0.174 | 0.3959 | No |
| 71 | ATP6V1F | ATPase H+ transporting V1 subunit F [Source:HGNC Symbol;Acc:HGNC:16832] | 3833 | 0.173 | 0.3991 | No |
| 72 | NDUFA6 | NADH:ubiquinone oxidoreductase subunit A6 [Source:HGNC Symbol;Acc:HGNC:7690] | 3834 | 0.173 | 0.4026 | No |
| 73 | ATP2B4 | ATPase plasma membrane Ca2+ transporting 4 [Source:HGNC Symbol;Acc:HGNC:817] | 3998 | 0.156 | 0.3931 | No |
| 74 | CLCN6 | chloride voltage-gated channel 6 [Source:HGNC Symbol;Acc:HGNC:2024] | 4138 | 0.143 | 0.3851 | No |
| 75 | ABCC3 | ATP binding cassette subfamily C member 3 [Source:HGNC Symbol;Acc:HGNC:54] | 4200 | 0.138 | 0.3832 | No |
| 76 | SLC12A5 | solute carrier family 12 member 5 [Source:HGNC Symbol;Acc:HGNC:13818] | 4308 | 0.128 | 0.3774 | No |
| 77 | ABCA12 | ATP binding cassette subfamily A member 12 [Source:HGNC Symbol;Acc:HGNC:14637] | 4451 | 0.117 | 0.3687 | No |
| 78 | ABCA8 | ATP binding cassette subfamily A member 8 [Source:HGNC Symbol;Acc:HGNC:38] | 4518 | 0.112 | 0.3658 | No |
| 79 | TAPBP | TAP binding protein [Source:HGNC Symbol;Acc:HGNC:11566] | 4527 | 0.111 | 0.3675 | No |
| 80 | SLC26A4 | solute carrier family 26 member 4 [Source:HGNC Symbol;Acc:HGNC:8818] | 4552 | 0.109 | 0.3678 | No |
| 81 | ABCB7 | ATP binding cassette subfamily B member 7 [Source:HGNC Symbol;Acc:HGNC:48] | 4669 | 0.099 | 0.3608 | No |
| 82 | ATP7A | ATPase copper transporting alpha [Source:HGNC Symbol;Acc:HGNC:869] | 4814 | 0.086 | 0.3513 | No |
| 83 | CYC1 | cytochrome c1 [Source:HGNC Symbol;Acc:HGNC:2579] | 4879 | 0.082 | 0.3479 | No |
| 84 | ABCC2 | ATP binding cassette subfamily C member 2 [Source:HGNC Symbol;Acc:HGNC:53] | 5206 | 0.056 | 0.3235 | No |
| 85 | COX6A2 | cytochrome c oxidase subunit 6A2 [Source:HGNC Symbol;Acc:HGNC:2279] | 5242 | 0.053 | 0.3219 | No |
| 86 | COX5A | cytochrome c oxidase subunit 5A [Source:HGNC Symbol;Acc:HGNC:2267] | 5329 | 0.045 | 0.3161 | No |
| 87 | ABCA2 | ATP binding cassette subfamily A member 2 [Source:HGNC Symbol;Acc:HGNC:32] | 5349 | 0.044 | 0.3155 | No |
| 88 | ATP1A3 | ATPase Na+/K+ transporting subunit alpha 3 [Source:HGNC Symbol;Acc:HGNC:801] | 5475 | 0.035 | 0.3064 | No |
| 89 | UQCRFS1 | "ubiquinol-cytochrome c reductase, Rieske iron-sulfur polypeptide 1 [Source:HGNC Symbol;Acc:HGNC:12587]" | 5509 | 0.032 | 0.3045 | No |
| 90 | ATP7B | ATPase copper transporting beta [Source:HGNC Symbol;Acc:HGNC:870] | 5751 | 0.012 | 0.2858 | No |
| 91 | CYB5A | cytochrome b5 type A [Source:HGNC Symbol;Acc:HGNC:2570] | 5850 | 0.005 | 0.2782 | No |
| 92 | ATP8A1 | ATPase phospholipid transporting 8A1 [Source:HGNC Symbol;Acc:HGNC:13531] | 6256 | -0.024 | 0.2470 | No |
| 93 | ATP13A2 | ATPase cation transporting 13A2 [Source:HGNC Symbol;Acc:HGNC:30213] | 6336 | -0.031 | 0.2414 | No |
| 94 | TOMM20 | translocase of outer mitochondrial membrane 20 [Source:HGNC Symbol;Acc:HGNC:20947] | 6339 | -0.031 | 0.2419 | No |
| 95 | ABCB9 | ATP binding cassette subfamily B member 9 [Source:HGNC Symbol;Acc:HGNC:50] | 6442 | -0.038 | 0.2347 | No |
| 96 | SLC4A8 | solute carrier family 4 member 8 [Source:HGNC Symbol;Acc:HGNC:11034] | 6455 | -0.039 | 0.2346 | No |
| 97 | ATP2A3 | ATPase sarcoplasmic/endoplasmic reticulum Ca2+ transporting 3 [Source:HGNC Symbol;Acc:HGNC:813] | 6559 | -0.046 | 0.2275 | No |
| 98 | ABCG4 | ATP binding cassette subfamily G member 4 [Source:HGNC Symbol;Acc:HGNC:13884] | 6574 | -0.047 | 0.2273 | No |
| 99 | TAP1 | "transporter 1, ATP binding cassette subfamily B member [Source:HGNC Symbol;Acc:HGNC:43]" | 6633 | -0.052 | 0.2239 | No |
| 100 | NDUFA5 | NADH:ubiquinone oxidoreductase subunit A5 [Source:HGNC Symbol;Acc:HGNC:7688] | 6789 | -0.062 | 0.2130 | No |
| 101 | ATP6V1B2 | ATPase H+ transporting V1 subunit B2 [Source:HGNC Symbol;Acc:HGNC:854] | 7074 | -0.086 | 0.1925 | No |
| 102 | ABCB8 | ATP binding cassette subfamily B member 8 [Source:HGNC Symbol;Acc:HGNC:49] | 7303 | -0.102 | 0.1767 | No |
| 103 | ABCG2 | ATP binding cassette subfamily G member 2 (Junior blood group) [Source:HGNC Symbol;Acc:HGNC:74] | 7473 | -0.116 | 0.1659 | No |
| 104 | SLC26A1 | solute carrier family 26 member 1 [Source:HGNC Symbol;Acc:HGNC:10993] | 7618 | -0.128 | 0.1572 | No |
| 105 | ABCA6 | ATP binding cassette subfamily A member 6 [Source:HGNC Symbol;Acc:HGNC:36] | 7655 | -0.131 | 0.1571 | No |
| 106 | ABCB11 | ATP binding cassette subfamily B member 11 [Source:HGNC Symbol;Acc:HGNC:42] | 7825 | -0.143 | 0.1468 | No |
| 107 | ATP6V1A | ATPase H+ transporting V1 subunit A [Source:HGNC Symbol;Acc:HGNC:851] | 8013 | -0.156 | 0.1354 | No |
| 108 | SLC1A1 | solute carrier family 1 member 1 [Source:HGNC Symbol;Acc:HGNC:10939] | 8157 | -0.167 | 0.1276 | No |
| 109 | ATP1A2 | ATPase Na+/K+ transporting subunit alpha 2 [Source:HGNC Symbol;Acc:HGNC:800] | 8218 | -0.173 | 0.1264 | No |
| 110 | ABCB1 | ATP binding cassette subfamily B member 1 [Source:HGNC Symbol;Acc:HGNC:40] | 8228 | -0.173 | 0.1293 | No |
| 111 | ATP1A1 | ATPase Na+/K+ transporting subunit alpha 1 [Source:HGNC Symbol;Acc:HGNC:799] | 8282 | -0.177 | 0.1288 | No |
| 112 | ABCD4 | ATP binding cassette subfamily D member 4 [Source:HGNC Symbol;Acc:HGNC:68] | 8652 | -0.205 | 0.1041 | No |
| 113 | CLCN7 | chloride voltage-gated channel 7 [Source:HGNC Symbol;Acc:HGNC:2025] | 8795 | -0.216 | 0.0974 | No |
| 114 | ABCA7 | ATP binding cassette subfamily A member 7 [Source:HGNC Symbol;Acc:HGNC:37] | 8856 | -0.222 | 0.0972 | No |
| 115 | ABCD3 | ATP binding cassette subfamily D member 3 [Source:HGNC Symbol;Acc:HGNC:67] | 8877 | -0.223 | 0.1003 | No |
| 116 | ABCD1 | ATP binding cassette subfamily D member 1 [Source:HGNC Symbol;Acc:HGNC:61] | 8930 | -0.226 | 0.1008 | No |
| 117 | ATP2A2 | ATPase sarcoplasmic/endoplasmic reticulum Ca2+ transporting 2 [Source:HGNC Symbol;Acc:HGNC:812] | 9015 | -0.233 | 0.0990 | No |
| 118 | ABCC5 | ATP binding cassette subfamily C member 5 [Source:HGNC Symbol;Acc:HGNC:56] | 9066 | -0.237 | 0.1000 | No |
| 119 | ABCC10 | ATP binding cassette subfamily C member 10 [Source:HGNC Symbol;Acc:HGNC:52] | 9218 | -0.250 | 0.0933 | No |
| 120 | SLC26A3 | solute carrier family 26 member 3 [Source:HGNC Symbol;Acc:HGNC:3018] | 9261 | -0.253 | 0.0952 | No |
| 121 | ABCC9 | ATP binding cassette subfamily C member 9 [Source:HGNC Symbol;Acc:HGNC:60] | 9346 | -0.259 | 0.0939 | No |
| 122 | KCNJ8 | potassium inwardly rectifying channel subfamily J member 8 [Source:HGNC Symbol;Acc:HGNC:6269] | 9421 | -0.266 | 0.0936 | No |
| 123 | SLC26A2 | solute carrier family 26 member 2 [Source:HGNC Symbol;Acc:HGNC:10994] | 9523 | -0.276 | 0.0914 | No |
| 124 | CYBRD1 | cytochrome b reductase 1 [Source:HGNC Symbol;Acc:HGNC:20797] | 9585 | -0.282 | 0.0924 | No |
| 125 | ABCB4 | ATP binding cassette subfamily B member 4 [Source:HGNC Symbol;Acc:HGNC:45] | 9807 | -0.302 | 0.0813 | No |
| 126 | ABCA1 | ATP binding cassette subfamily A member 1 [Source:HGNC Symbol;Acc:HGNC:29] | 9874 | -0.309 | 0.0824 | No |
| 127 | SLC26A6 | solute carrier family 26 member 6 [Source:HGNC Symbol;Acc:HGNC:14472] | 9898 | -0.311 | 0.0870 | No |
| 128 | ATP13A1 | ATPase 13A1 [Source:HGNC Symbol;Acc:HGNC:24215] | 9939 | -0.315 | 0.0904 | No |
| 129 | ATP4A | ATPase H+/K+ transporting subunit alpha [Source:HGNC Symbol;Acc:HGNC:819] | 10417 | -0.363 | 0.0605 | No |
| 130 | ATP2C1 | ATPase secretory pathway Ca2+ transporting 1 [Source:HGNC Symbol;Acc:HGNC:13211] | 10432 | -0.364 | 0.0669 | No |
| 131 | ABCA4 | ATP binding cassette subfamily A member 4 [Source:HGNC Symbol;Acc:HGNC:34] | 10554 | -0.379 | 0.0652 | No |
| 132 | ATP2C2 | ATPase secretory pathway Ca2+ transporting 2 [Source:HGNC Symbol;Acc:HGNC:29103] | 10580 | -0.382 | 0.0710 | No |
| 133 | CFTR | CF transmembrane conductance regulator [Source:HGNC Symbol;Acc:HGNC:1884] | 10920 | -0.420 | 0.0531 | No |
| 134 | ATP13A3 | ATPase 13A3 [Source:HGNC Symbol;Acc:HGNC:24113] | 11136 | -0.448 | 0.0455 | No |
| 135 | ABCC1 | ATP binding cassette subfamily C member 1 [Source:HGNC Symbol;Acc:HGNC:51] | 11462 | -0.497 | 0.0302 | No |
| 136 | TAP2 | "transporter 2, ATP binding cassette subfamily B member [Source:HGNC Symbol;Acc:HGNC:44]" | 11616 | -0.519 | 0.0289 | No |
| 137 | ATP2B1 | ATPase plasma membrane Ca2+ transporting 1 [Source:HGNC Symbol;Acc:HGNC:814] | 11648 | -0.524 | 0.0373 | No |
| 138 | ABCA5 | ATP binding cassette subfamily A member 5 [Source:HGNC Symbol;Acc:HGNC:35] | 11884 | -0.568 | 0.0305 | No |
| 139 | MT-ND5 | mitochondrially encoded NADH:ubiquinone oxidoreductase core subunit 5 [Source:HGNC Symbol;Acc:HGNC:7461] | 12743 | -0.906 | -0.0181 | No |
| 140 | SLC26A10 | solute carrier family 26 member 10 [Source:HGNC Symbol;Acc:HGNC:14470] | 12899 | -1.475 | 0.0001 | No |
Table: GSEA details [plain text format]

  

Fig 2: GOMF\_PRIMARY\_ACTIVE\_TRANSMEMBRANE\_TRANSPORTER\_ACTIVITY      
 Blue-Pink O' Gram in the Space of the Analyzed GeneSet

  

Fig 3: GOMF\_PRIMARY\_ACTIVE\_TRANSMEMBRANE\_TRANSPORTER\_ACTIVITY: Random ES distribution      
 Gene set null distribution of ES for **GOMF\_PRIMARY\_ACTIVE\_TRANSMEMBRANE\_TRANSPORTER\_ACTIVITY**
